# Evaluating anti-Orthopoxvirus antibodies in individuals from Brazilian rural areas prior to the bovine vaccinia era

**DOI:** 10.1590/0074-02760150215

**Published:** 2015-09

**Authors:** Poliana de Oliveira Figueiredo, André Tavares da Silva-Fernandes, Bruno Eduardo Fernandes Mota, Galileu Barbosa Costa, Iara Apolinário Borges, Paulo César Peregrino Ferreira, Jônatas Santos Abrahão, Erika Martins Braga, Erna Geessien Kroon, Giliane de Souza Trindade

**Affiliations:** 1 Universidade Federal de Minas Gerais, Instituto de Ciências Biológicas, Departamento de Microbiologia, Laboratório de Vírus, Belo Horizonte, MG, Brasil; 2 Universidade Federal de Minas Gerais, Instituto de Ciências Biológicas, Departamento de Parasitologia, Laboratório de Malária, Belo Horizonte, MG, Brasil

**Keywords:** Orthopoxvirus, smallpox vaccine, bovine vaccinia, retrospective serosurvey

## Abstract

Vaccinia virus naturally circulates in Brazil and is the causative agent of a zoonotic disease known as bovine vaccinia (BV). We retrospectively evaluated two populations from the Amazon and Southeast Regions. BV outbreaks had not been reported in these regions before sample collection. Neutralising antibodies were found in 13 individuals (n = 132) with titres ranging from 100 ≥ 6,400 neutralising units/mL. Univariate analysis identified age and vaccination as statistically significant risk factors in individuals from the Southeast Region. The absence of detectable antibodies in vaccinated individuals raises questions about the protection of smallpox vaccine years after vaccination and reinforces the need for surveillance of Orthopoxvirus in Brazilian populations without evidence of previous outbreaks.

Thirty-four years ago, the world celebrated the eradication of smallpox, a lethal disease caused by Variola virus infection. The massive antismallpox vaccination campaign was promoted by the World Health Organization (WHO) during the 1960’s and 1970’s (Fenner et al. 1988, Damon 2013). Vaccinia virus (VACV), a species belonging to the Orthopoxvirus (OPV) genus that demonstrates serological cross-reactivity with other OPV species, was used as the vaccine antigen during the WHO campaign. Following smallpox eradication in the late 1970’s, vaccination was suspended due to several instances of adverse reactions to the vaccine (Cono et al. 2003).

The natural circulation of VACV began to be reported in Brazil in 1999 and has been associated with several exanthematic VACV outbreaks that have been described in Brazilian rural areas (da Fonseca et al. 2011,Kroon et al. 2011, Singh et al. 2012, Shchelkunov 2013). VACV infection causes lesions on the teats and udders of dairy cattle, leading to a decrease in milk production. VACV is the cause of a zoonotic disease known as bovine vaccinia (BV) and can be transmitted to humans by direct contact with infected animals during milking, resulting in lesions on the hands and arms (Damaso et al. 2000, Trindade et al. 2003, 2007, 2009, Leite et al. 2005, Lobato et al. 2005, Megid et al. 2008, Silva-Fernandes et al. 2009, Abrahão et al. 2010a, Schatzmayr et al. 2011, deAssis et al. 2013, de Sant’Ana et al. 2013). The lesions evolve from macules to papules to vesicles to pustules, which ulcerate and result in scar formation. Nonspecific symptoms such as fever and lymphadenopathy can also be observed in most infected individuals (Silva-Fernandes et al. 2009, Trindade et al. 2009). The transmission of VACV is associated with unprotected contact between BV-affected cattle and milkers.

Although BV outbreaks associated with vaccine strains were reported during the smallpox eradication campaigns in Latin America and Asia (Fenner et al. 1988), these notifications ceased after vaccination suspension, with only a few cases reported in the 1980’s in Southeast Brazil related to contact with cows during milking (Silva et al. 1986). It remains unclear why BV outbreaks have re-emerged after 20 years of absence. Possible explanations for the lack of reported cases for decades include the effective immune response generated by massive smallpox vaccination during the 1970’s, significant under-reporting leading to misdiagnoses and the absence of a specific government-enforced surveillance policy (Trindade et al. 2009, da Fonseca et al. 2011). Despite the fact that these outbreaks, as well as the individuals affected by each case, seem to be systematically increasing from year to year both in quantity and in geographic distribution, there remains no officially reported number of human cases across the country.

Theories that propose VACV circulation and maintenance in Brazilian forests have gained attention in recent years, mainly after the detection of VACV in wild and peridomestic animals (Abrahão et al. 2009, 2010b, Peres et al. 2013). Indeed, VACV strains were previously detected in wild and sentinel rodents from the Brazilian Amazon and southeastern forests in the 1960’s and 1970’s (Lopes et al. 1965, Fonseca et al. 1998). Thus, human exposure to VACV could be related to activities distinct from milking, as suggested by Mota et al. (2010).

Although there are numerous studies related to the occurrence of VACV in Brazil, little is known about anti-OPV immunity in vulnerable populations. A recent study performed by our research group identified a low prevalence of OPV immunity in laboratory workers (Costa et al. 2013). However, most studies have concentrated their efforts on the analysis of humoral responses in patients affected by BV outbreaks or in rural areas where the occurrence of BV has never been reported (Silva-Fernandes et al. 2009, Mota et al. 2010). Indeed, there are no data thus far regarding humoral immunity to OPV in rural populations at high risk of VACV infection.

The present study retrospectively analysed serological protection against OPV in two Brazilian populations from the Amazon and Southeast Regions, where BV cases have not been observed since the late 1990’s. Our results raise interesting questions regarding VACV circulation in Brazil in the period preceding the onset of the 1999 BV outbreaks and the levels of protection in these populations today.

We analysed 62 sera samples from the municipality of Mantena, in the state of Minas Gerais (18º46’55’S 40º58’48’’W) and 70 sera samples from the municipality of Terra Nova do Norte, in the state of Mato Grosso (10º31’01’’S 55º13’51’’W) (Figure). These samples were collected between 1995-1996 as part of a malaria field investigation (Braga et al. 1997, 2002). Ethical approval for this study was granted by the Research Ethical Committee at the Federal University of Minas Gerais under registration protocol FR-413704. Written informed consent was obtained from all study participants or from their parents (Braga et al. 1997, 2002).

Plaque reduction neutralization tests (PRNT) were run with VACV-Western Reserve (WR) strains as controls, as previously described by Newman et al. (2003) with modifications and recently by our team (Costa et al. 2013). Briefly, serum samples were heated to 56ºC for 30 min to denature complement system proteins and then diluted in Eagle’s minimum essential medium (MEM) free of foetal bovine serum (FBS) to a screening ratio of 1:40. Samples were added to the same volume (1:1) of a solution containing approximately 100 plaque-forming units of VACV-WR. This mixture was incubated for 16 h at 37ºC. Six-well plates containing BSC-40 cells monolayers were inoculated with the mixture. Plates were incubated at 37ºC for 1 h in an atmosphere with 5% CO_2_. MEM supplemented with 2% FBS were added to each well and incubated again at 37ºC with 5% CO_2_ for 48 h. Cell monolayers were fixed with 10% formalin and stained with 1% crystal violet solution. A sample that inhibited 50% of plaque formation was considered positive for neutralising antibodies. Samples were tested in triplicate and all positives were titrated.

**Figure:**
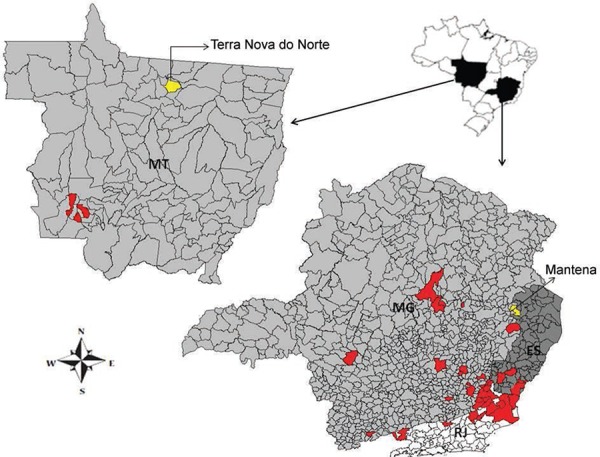
Regions with bovine vaccinia notification after 1995 (red). Brazilian states: Espírito Santo (ES), Minas Gerais (MG), Mato Grosso (MT), Rio de Janeiro (RJ).

Epidemiological information, such as gender, age and occupation, were also available. These data were converted into variables and tested to assess their relationship to the presence or absence of neutralising antibodies. Eight samples from Terra Nova do Norte were missing epidemiological information; thus, only 62 samples were considered for statistical analysis. Data were collected using the open access software EpiData (provided by the Pan-American Health Organization) and analysed using chi-square or Fisher’s exact tests, as appropriate.

The overall seroprevalence of anti-OPV neutralising antibodies was 9.84% (13 individuals from the 132 tested) with titres ranging from 100 ≥ 6400 neutralising units/mL. This figure was three times lower when compared with the prevalence of 27.89% observed in the Amazon Region (82 individuals from 294 tested) (Mota et al. 2010) and much lower when compared to studies performed in African regions (Lederman et al. 2007, Reynolds et al. 2010). However, our survey showed a high prevalence in relation to a study conducted in Sierra Leone (MacNeil et al. 2011). All previous studies used ELISA tests to detect anti-OPV antibodies. ELISA is a biochemical test that detects all antibodies by class, while the PRNT is a biological test that detects specific neutralising antibodies and is considered the gold standard approach for serological diagnosis (Storch & Wang 2013).

In addition to these technical differences, other factors that may have contributed to differences in observed prevalence rates include cultural and behavioural differences for risk/exposure factors for OPV infection and patterns of virus circulation in the regions studied. In Brazil, OPV infections are related to outbreaks affecting dairy cattle (Damaso et al. 2000, Trindade et al. 2003, 2007,2009, Leite et al. 2005, Lobato et al. 2005, Megid et al. 2008, Silva-Fernandes et al. 2009, Abrahão et al. 2010a, Schatzmayr et al. 2011, deAssis et al. 2013, de Sant’Ana et al. 2013) and laboratory activities (Costa et al. 2013). In Africa, exposure to OPV is mainly related to hunting for food, working and living close to sylvan animals, passing through forested areas, as well as secondary human transmission (person-to-person transmission between family members living in the same household) (Reynolds et al. 2010, Damon 2011).

The seropositive individuals found in this study had been vaccinated and included six males and seven females. Nine of these individuals were from Mantena. The median age in both locations was 27 years and ranged from 11-64 years in Terra Nova do Norte and from eight-76 years in Mantena. Summary statistics such as demographic data, occupation and vaccination status by regional population are shown in Tables I, II. Statistical analyses showed significant associations when neutralising antibodies were grouped by age and vaccination status (Table I).

**TABLE I:** Analysis of the characteristics of the population of Mantena, state of Minas Gerais, Brazil, according to the seropositivity for anti-Orthopoxvirus neutralising antibodies

Variables	Tested individuals n (%)^*a*^	PRNT positives n (%)	PRNT negatives n (%)	p^*b*^
Gender				
Male	31 (50)	5 (16.1)	26 (83.9)	1.000
Female	31 (50)	4 (12.9)	27 (87.1)	
Age (years)				
≤ 18	19 (30.6)	0 (0)	19 (100)	0.006
19-26	12 (19.4)	1 (8.3)	11 (91.7)	
27-36	11 (17.8)	6 (54.5)	5 (45.5)	
> 36	20 (32.2)	2 (10)	5 (45.5)	
Vaccination status				
Yes	42 (67.8)	9 (21.5)	33 (78.5)	0.047
No	20 (32.2)	0 (0)	20 (100)	
Total	62 (100)	9 (14.5)	53 (85.5)	

*a*: frequency of tested individuals per category;*b*: Fisher’s exact test; PRNT: plaque reduction neutralization test.

**TABLE II:** Analysis of the characteristics of the population of Terra Nova do Norte, state of Mato Grosso, Brazil, according to the seropositivity for anti-Orthopoxvirus neutralising antibodies

Variables	Tested individuals n (%)^*a*^	PRNT positives n (%)	PRNT negatives n (%)	p^*b*^
Gender				
Male	40 (57.2)	1 (16.1) 0 (0)	39 (83.9)	0.291
Female	25 (35.7)	3 (12.9)	22 (87.1)	
No data	5 (7.1)	0 (0)	5 (100)	
Age (years)				
≤ 18	11 (15.7)	0 (0)	11 (100)	0.299
19-26	18 (25.7)	2 (11.1)	16 (88.9)	
27-36	16 (22.9)	2 (12.5)	14 (87.5)	
> 36	18 (25.7)	0 (0)	18 (100)	
No data	7 (10)	0 (0)	7 (100)	
Vaccination status				
Yes	48 (68.6)	4 (8.3)	44 (91.7)	0.564
No	15 (21.4)	0 (0)	15 (100)	
No data	7 (10)	0 (0)	7 (100)	
Occupation				
Gold miner	17 (24.3)	0 (0)	17 (100)	0.618
Farmer	27 (38.6)	2 (7.4)	25 (92.6)	
Others	20 (28.5)	2 (10)	18 (90)	
No data	6 (8.6)	0 (0)	6 (100)	

Total	70 (100)	4 (5.7)	66 (94.3)	

*a*: frequency of tested individuals per category;*b*: Fisher’s exact test; PRNT: plaque reduction neutralization test.

The higher proportion of vaccinated individuals without neutralising antibodies in both populations (n = 77; 85.5%) could be explained by a myriad of factors which may be unrelated to vaccination, as this was a retrospective study and the subjects under investigation were chosen in the context of a study on malaria infection and circulation (Braga et al. 1997, 2002). Thus, when samples were obtained, no efforts were made to look specifically for subjects with clinical indications of previous OPV infection or to determine if the individuals had been successfully vaccinated, which is known to show a strong correlation with smallpox vaccination. Additionally, as discussed by Fenner et al. (1988), there was variable efficacy in the smallpox vaccines produced in Brazil. The distribution channels throughout different Brazilian geographical areas, in addition to inadequate transportation systems and cold chains, sometimes failed to meet accepted quality standards.

Many authors have demonstrated that anti-OPV immunity persists for decades (Hammarlund et al. 2003, Hatakeyama et al. 2005, Kim et al. 2007, Taub et al. 2008). Indeed, high anti-OPV neutralising antibody titres and statistically significant associations between age (p = 0.0006) and vaccination status (p = 0.047) were found in individuals from Mantena (Table I). This finding might be explained by the quality of vaccine or quantity of doses received, although maintenance of the cold chain may have been broken before the smallpox vaccine reached rural populations residing in places with difficult access (Fenner et al. 1988). On the other hand, the high titres found in this population could also suggest that human exposure to OPV in these regions may have occurred prior to the BV era, indicating silent VACV circulation and exposure. Indeed, a previous study demonstrated anti-OPV immunity in Brazilian rural populations in the absence of VACV outbreaks (Mota et al. 2010).

Several factors can contribute to the presence of antibodies in response to OPV infection (Lederman et al. 2007, Kennedy et al. 2009, Mota et al. 2010, Reynolds et al. 2010, Costa et al. 2013). In our statistical analyses, occupation did not enhance the risk of OPV exposure or the presence of neutralising antibodies in individuals from Terra Nova do Norte, despite the fact that farmers (mainly cattle handlers) are directly affected by BV (Trindade et al. 2003, 2007, 2009, Leite et al. 2005, Lobato et al. 2005, Megid et al. 2008, Silva-Fernandes et al. 2009, Abrahão et al. 2010a, Schatzmayr et al. 2011, deAssis et al. 2013, de Sant’Ana et al. 2013).

In conclusion, knowledge of increasing OPV infections and the discovery of novel zoonotic OPV (Vora et al. 2015) pose a continuous and growing threat to human health and information on their epidemiologic features is important in order to prevent new outbreaks. Indeed, VACV seroprevalence studies in Brazil are scarce and most studies conducted in the country to date have focused on outbreak investigation. Furthermore, the absence of neutralising antibodies in vaccinated individuals found in this study and the current occurrence of VACV in all Brazilian territories reinforces the need for OPV surveillance regardless of known outbreaks. Our findings also highlight the need to strengthen global surveillance of OPV infections in both humans and animals (Shchelkunov 2013, Vora et al. 2015). Additional epidemiological studies are ongoing that will further contribute to our understanding of OPV epidemiology by elucidating OPV-vulnerable populations and characterising OPV silent circulation in the absence of outbreaks.
